# Institutional Notes and News

**Published:** 1909-11-27

**Authors:** 


### Institutional Notes and News.

### HOSPITAL FOR INVALID GENTLEWOMEN.

This hospital, lately situated at 90 Harley Street, was originally organised and under the superintendence of Miss Florence Nightingale. Miss Nightingale asks and prays her "friends who still remember her not to let this truly sacred work languish and die from want of a little more money." Owing to the expiration of the lease £18,000 is required to erect and equip a new building, which will shortly be opened at 19 Lisson Grove. Only £8,000 of this sum is still required, and as this hospital is the only one which provides medical and surgical attendance with nursing for gentlewomen of limited means, we hope that the Treasurer, Mr. Bridgman, M.P., of 13 Mansfield Street, W., may speedily receive the relatively small sum now required to complete the building fund.

### THE CUMBERLAND INFIRMARY.

The Governors have decided to allocate the sum of £6,235, which they have received during the past few years in "unrestricted" legacies, to the Building Fund, thus bringing it up to a little over £25,000, or within £5,000 of the amount which is required to complete the scheme for enlarging and modernising the institution. The need of applying legacies, which should ordinarily be invested in revenue-producing stocks, to capital purposes is to be regretted, but it is imperative. Money is required to carry on the work, and the Governors are compelled to turn to the only quarter where for the moment it is obtainable. Their hope that they would be able to raise sufficient in special subscriptions to carry through the scheme of extension was, events have proved, too sanguine. Little more than one half of the necessary money has as yet been obtained in donations.

### BARRY VOLUNTARY HOSPITAL.

A sale of work in aid of the funds of the Voluntary Hospital for the Destitute Sick and Dying, Holton Road, Barry Docks, was opened by Lady Llangattock on Wednesday, October 27. The attractions included stalls of work, plain and fancy goods, *x*-ray demonstrations, refreshments, bran tub, music, etc. In opening the bazaar Lady Llangattock said the Voluntary Hospital for the Destitute Sick and Dying was doing splendid work in the district. Since the hospital was opened in 1898, over 1,286 men, women, and children had been treated. Nearly half the patients treated were sailors. Kind and sympathetic friends from many parts of the world had sent help, and donations had come from various parts of the British Isles, India, America, Germany, France, Italy, and Switzerland, to help pay off the debt on the building. The sum of £643 was wanted to clear off the debt, but a great effort was to be made to account for it this year.

### SOUTH DEVON AND EAST CORNWALL HOSPITAL.

The Committee is making a special appeal on behalf of the Institution. The appeal points out that the growth of population and, still more, the increasing appreciation of the benefits that hospital treatment can alone secure have attracted an ever-increasing number of patients. Up to 1902 the number of occupied beds was rarely more than 100; at the present time the average is 140. A large proportion of the cases admitted are of the more serious character, requiring the treatment which only a large and fully equipped hospital can supply, other cases being dealt with by smaller hospitals which are doing excellent work in various localities. These graver cases of disease and injury are obviously by far the most expensive. The cost of maintaining the hospital is now something like £10,000 a year, though there is strict economy of management, and the Committee exercise careful and constant scrutiny into every item of expenditure. Unless increased help is forthcoming the Committee will be compelled to close at least two large wards of the hospital. The two forms of assistance which the Committee earnestly solicits are (1) new or increased annual subscriptions; (2) contributions to pay off the adverse balance of £3,500.

### COVENTRY HOSPITAL SATURDAY FUND.

Mr. J. A. Rudd, the secretary of the Coventry and Warwickshire Hospital, Coventry, writes: For the information of your readers, I am pleased to state that the amount received from the Hospital Saturday Fund Committee in Coventry this year amounted to £2,900. This constitutes a record for the Hospital Saturday movement in Coventry, the last highest amount attained being £2,825. This record speaks highly of the generosity of the working classes in this city and district, and is an admirable testimonial to this organisation of working men in collecting such a large sum, especially when it is taken into consideration that the amount is collected weekly in the smaller coins of the realm.

### WOMEN'S HOSPITAL, SOHO.

The Hospital for Women, Soho Square, W., which was founded in 1842 by Dr. Protheroe Smith, and which has carried on its good work for the past sixty years in the hospital buildings at its disposal, is now in process of rebuilding. The new hospital, which will contain 67 beds, as against 60 in the old building, will be finished in 1910 and will be a thorougly up-to-date institution fitted with the latest appliances of medical science. The entire cost will be about £18,000, of which £12,000 has already been received. About £6,000 is now required to complete the work, of which King Edward's Hospital Fund has promised £3,000 on condition that the remaining £3,000 is raised by March 1910. To attain this end a large bazaar and entertainment, to be held early in 1910, is being organised by a committee of influential ladies. Contributions to the Rebuilding Fund are earnestly solicited and will be gratefully acknowledged by the Secretary, Mr. Alfred Hayward.

### THE QUEEN'S HOSPITAL FOR CHILDREN.

A special meeting of the clergy and laity of all denominations in the East-end was held at the Queen's Hospital for Children, Hackney Road, Bethnal Green, on Tuesday, to decide what means should be taken to help the hospital out of a press of difficulties. If half the wards, 62 beds, are not to be shut down by the end of the year £4,000 must be raised immediately. The deficit on the maintenance fund is now £5,500, and there is a debt on mortgage of £5,370. The Rev. J. E. Watts-Ditchfield, who presided, pointed out that the closing of beds in the hospital to-day, when the demands for accommodation in both in-patient and out-patient departments are increasing, would be nothing less than disastrous to the neighbourhood. The meeting resolved itself into an emergency appeal committee, adopted a resolution appealing for £4,000, and considered a variety of suggestions for the immediate raising of money. The important co-operation of the clergy of all denominations was obtained.

### HANDEL COSSHAM MEMORIAL.

The Handel Cossham Memorial Hospital, Kingswood, Bristol, which was closed during the epidemic of small-pox in the city and neighbourhood, was opened again on November 10. The hospital has been closed, in fact, till money was forthcoming from the Chancery authorities to meet the expenses of its existence. Now that the money has been obtained, a staff has been appointed, but only two of the four wards have yet been reopened.

### GREAT NORTHERN CENTRAL.

The Great Northern Central Hospital, Holloway Road, is in such urgent need of funds that the management has no alternative to the closing of at least 50 of its 179 beds at the end of the year, unless £12,000 is guaranteed at an early date. To put the claims of the hospital prominently before the public with a view to make it possible to clear off its debt and carry on its work in a very poor district, a festival banquet was held at the Whitehall Rooms, Hôtel Métropole, on Wednesday, November 24, Sir Edgar Speyer presiding.

### BRIGHTON THROAT AND EAR HOSPITAL.

One of the special features of the year has been the reduction of the bank loan from £1,200, at which amount it has remained since the year 1906, to £750. At the beginning of the year Mr. R. J. Messent, L.D.S., R.C.S., was elected honorary dental surgeon, in place of Mr. W. R. Wood, who resigned after having held the post since 1902. The Committee regret extremely the death of one of their number, Mr. Arnold Chipperfield, who rendered great assistance to the hospital for many years, and was a generous contributor to its finances. The Ladies' Linen League, under the able superintendence of the matron and its honorary secretary, Miss Scatliff, have again supplied the linen required in the hospital.

### METROPOLITAN HOSPITAL.

The hospital, which has been closed since June for the purpose of bringing it up to date, was reopened last week. The cost of the improvements is £10,500, and towards this only £6,500 (£4,000 of which represents stock sold, and therefore a decrease in income) has been raised so far. The operating theatre has been entirely remodelled, and an anæsthetic room, a steam sterilising plant, and other modern appliances added. New pathological laboratories for research work have been put in, while the electrical and massage department, which includes the *x*-ray and high-frequency installation, has been brought up to date. The drainage system has been completely relaid, and baths and other sanitary appliances have been renewed throughout. The boiler-room has been reconstructed, and by a new device it has been found possible to make a saving of two tons of coal a week. The appearance of the interior of the building has been greatly improved, the whole wall area, which was originally bare brick, having been plastered and decorated, while bath-rooms, kitchens, etc., have been tiled throughout. The out-patients' department has been considerably enlarged, with new surgeries and waiting-rooms.

### CONDEMNING A HOSPITAL.

A special meeting of the Hinckley Joint Hospital Committee was held last week to consider the question of the erection of permanent buildings in place of the present hospital, which is inadequate, especially for patients in the convalescent stage. The Medical Officer of Health for the district stated that the hospital is in a dilapidated and insanitary state throughout. There is not even a supply of hot water, and it is ridiculously understaffed. He recommended that a building be erected to accommodate six cases of diphtheria, six cases of typhoid fever, and ten cases of scarlet fever, and also that it be supplied with the telephone.

Dr. Robinson said the building is not fit for hospital purposes, and quite agreed with Dr. O'Connor's suggestions for a new building. Such a hospital would cost something like £4,500, including architect's fees. If they had an efficiently-equipped place the County Council would make them a grant of half of the establishment expenses, not exceeding £10 per bed for the period of which the loan extended, and one-sixth the establishment expenses after that time. In the case of Melton Mowbray they put up a building at a cost of £8,024, and the County Council made them a grant of £200 per annum.

Mr. Biggs moved, and Mr. Hopcroft seconded, that the committee, having heard the views expressed by Dr. Robinson, agrees to give support to those views, and recommends that steps be taken to carry them into effect. This was agreed to.

---

### IS A HOSPITAL LAUNDRY A FACTORY?

A curious question, which appears to be of considerable interest to managers and governors of hospitals, was raised and decided before the Reading Justices on October 8. A full report of the proceedings appears in the *Justice of the Peace*, November 6, 1909, but the material facts are comparatively simple. It appears that the governors and secretary of the Royal Berks Hospital were summoned by the Home Office for (*a*) not affixing the prescribed abstract of the Act required by Sec. 128 of the Factory and Workshop Act, 1907; and (*b*) for not keeping a general register pursuant to Sec. 129 of that Act. It sounds extraordinary to think of a hospital being called a laundry under any stretch of the legal imagination, but the question related to the hospital laundry.

In 1907 an act was passed amending the Factory Act in so far as it relates to laundries. By Sec. 1 of this Act the Factory Act of 1901 is made to apply to "laundries carried on by way of trade or for the purpose of gain, or carried on as ancillary to another business or incidentally to the purposes of any public institution." It was contended on the part of the Home Office that the Berks Hospital is a public institution within the meaning of this provision. In support of this it was pointed out that the rules provide that the hospital shall be open for the reception and treatment of sick and disabled poor of all countries, and, further, that there is a Workpeople's Hospital Association, the members of which are entitled to be treated in the hospital free of charge. It was also agreed or admitted that a steam laundry is carried on in connection with the hospital; that the hospital is open for the reception and treatment of the sick and disabled poor of all countries; and that in cases of accident the resident medical officers are bound to receive the patients without delay or recommendation. It was contended on the part of the hospital that it is not a public institution, because in reality all patients regularly treated have to be received either on the recommendation of subscribers or as members of the Workpeople's Association. In the event the magistrates held that the hospital is a public institution, and the unfortunate hospital was fined the minimum fine of 5s., with £5 5s. costs. So far as one can see there is nothing to distinguish the Royal Berks from any other hospital. The result of the statute above mentioned and of this decision is that the laundries of all these institutions are factories within the meaning of the Act of 1907.

